# Interrogating Healthy Community Discourse in Municipal Policies: Priorities of a Medium-Sized CMA in Ontario, Canada

**DOI:** 10.3390/ijerph22020172

**Published:** 2025-01-27

**Authors:** Keely Stenberg, Jennifer Dean

**Affiliations:** School of Planning, University of Waterloo, Waterloo, ON N2L 3G1, Canada; jennifer.dean@uwaterloo.ca

**Keywords:** healthy communities, health in all policies, social determinants of health, political will, policy analysis

## Abstract

The World Health Organization’s *Healthy Cities* movement recommends action on the determinants of health and health equity. While economic and ecological circumstances have been studied with respect to health outcomes, research shows that the relationship between these broad determinants and population health is not always clear. Municipal governments, whose relative proximity to individuals means that they are optimally situated to address local health concerns, can demonstrate political will for healthy communities by developing health community policies. Therefore, the aim of this study is to interrogate how the idea of a ‘healthy community’ has been conceptualized by municipal governments in order to inform the future uptake of the concept. This study uses a post-structural policy analysis to examine government discourse on healthy communities in a medium-sized census metropolitan area (CMA) in Ontario, Canada. The findings highlight economic growth and ecological sustainability as priorities for fostering a healthy community. With emphasis on long-standing issues linking health outcomes to broader societal conditions, this study calls on municipal governments to explicitly consider the health impacts of healthy community strategies and adoption of a Health-in-All-Policies (HiAP) approach.

## 1. Introduction

### 1.1. Healthy Cities Movement

The first International Conference on Health Promotion and the Ottawa Charter for Health Promotion highlighted the need to take action on the determinants of health through a health promotion lens [1]. Building on these calls for action, the World Health Organization (WHO) implemented the *Healthy Cities* program in 1986. According to the WHO, healthy cities are created through multiple complementary drivers: (1) taking action on the social determinants of health; (2) considering equity in initiative development; (3) supporting intersectoral collaboration in and outside of the healthcare sector; (4) involving citizens and residents in decision making; and (5) requiring political will and action [2]. Underlying these drivers is the need for institutions and governments to make broad social changes that support the health of the population. Further, the *Healthy Cities* initiatives are tailored to the local context, which requires stakeholders to be attentive to community needs.

Although the WHO’s *Healthy Cities* movement has its roots in Europe, initiatives have been implemented in the United States [3], Canada [4,5,6], Korea [7], China [8] and elsewhere. As an early participant in the movement, there have been multiple healthy city/community initiatives launched over the past 35 years across Canada. Examples of these initiatives include the 1987 *Canadian Healthy Communities Project* developed in partnership between the Canadian Institute of Planners, Canadian Public Health Association, and Federation of Canadian Municipalities [6]; the 2009 *Healthy Canada by Design* project implemented through the Coalitions Linking Action & Science for Prevention (CLASP) program [9]; the 2019 *Healthy Cities Research Training Platform* supported in partnership by the Canadian Institutes of Health Research (CIHR), the Natural Sciences and Engineering Research Council (NSERC), and the Social Sciences and Humanities Research Council (SSHRC) [10]; and the 2021 *Canada Healthy Communities Initiative* funded by the Government of Canada [4]. Reflecting the cornerstone principles of the *Healthy Cities* movement, several municipal governments in Canada have developed their own healthy community strategies, including Kelowna [11] and Vancouver [12,13] in British Columbia; Kitchener [14] and Mississauga [15] in Ontario; and St. John’s in Newfoundland [16]. However, despite the widespread participation in Canada, there lacks an understanding of what health concerns and determinants of health municipal governments are targeting through their healthy community policies. In order to address this gap, this paper first explores the literature on the socioeconomic and ecological determinants of population health and the concept of Health-in-All-Policies (HiAP), before critically exploring how a region mandated to create healthy communities is addressing this responsibility.

### 1.2. Socioeconomic and Ecological Determinants of Health

The conditions in which individuals and populations live have long been recognized as determinants of health. Socioeconomic and ecological conditions can be identified as distal determinants of health [17,18] or the “‘Causes of the causes’ of illness” [18], p. 302. While economic growth does not necessarily translate into improved population health outcomes [19,20], broad economic conditions can act directly and indirectly (i.e., with/through other determinants) to influence health [17]. For example, discretionary government fiscal policy such as taxation approaches and spending commitments on social services and basic needs have been recognized as determinants of population health [17,21]; however, in their assessment of the impact of policies on socioeconomic determinants of health in high Organisation for Economic Co-operation and Development (OECD) countries, Mosquera et al. [21] found that the impacts of discretionary fiscal policy are context-specific. That is, the health impacts of policies promoting economic growth are highly sensitive to the local context, a finding that was recently echoed by a study on economic activity at the national level [19]. As such, how governments spend the additional economic resources they generate matters to health and well-being [20,22].

With respect to more proximate determinants of health, socioeconomic status and social class have been assessed using a variety of factors, such as education, income, and occupation [23]. In their review, Barakat and Konstantinidis [23] found that increases in individual socioeconomic status was positively correlated with mental health, while a decrease in socioeconomic status was linked to an increased risk of cardiovascular disease. The authors suggest that developing policies that prioritize individuals who experience socioeconomic disadvantages, especially when exacerbated by other forms of inequality based on ethnicity and gender, could improve physical and mental health, and overall well-being. Similarly, Moor et al.’s [24] systematic review of factors linking socioeconomic inequalities and self-rated health found that material factors have a greater direct impact on self-rated health and also indirectly effect psychosocial and behavioural circumstances. These authors recommended that policies targeted at improving material conditions, such as economic, working, and living conditions, would reduce social inequalities and inequities in self-rated health. Therefore, in order to create healthy communities through action on the determinants of health, municipal governments must address issues specific to the local context related to individual and population economic conditions, thereby ensuring that financial and physical resources are available to support the health and well-being of all residents.

Recently, discussion of the *Healthy Cities* movement has identified ecological determinants of health as a pressing priority for North America [25]. Human activities and a reliance on fossil fuels have resulted in the increased production of greenhouse gases, leading to global warming [26]. The impacts of global warming and climate change are now widely studied [27,28], including the physical and mental health implications of extreme weather events (e.g., drought, floods, and wildfires), poor air and water quality, and increasing temperatures and humidity [28,29,30]. In particular, air quality and temperature are the most commonly studied ecological determinants, which are linked to several health outcomes (e.g., mortality, and cardiovascular and respiratory diseases) [28]. To protect global health in an era of climate change, several mitigation and adaption measures have been proposed to manage greenhouse gas emissions in communities [26,31]. Prominent municipal mitigation techniques include changes to transportation practices (i.e., using higher-efficiency vehicles and public transportation) [26], ‘greening’ buildings (e.g., through the use of solar power) [26], and managing waste (e.g., by recycling) [26,31]. Reinforcing these findings, Luyten et al.’s [27] scoping review of the health impacts of climate mitigation and adaptation strategies identified that the energy (33%), transportation (32%), and building (15%) sectors as most often the target for mitigation efforts while infrastructure makes up the majority (56%) of adaptation measures.

### 1.3. The Health-in-All-Policies Approach

Advocacy related to individual- and population-level health outcomes is evident in calls from researchers for policy-makers to take action on social determinants [32] by adopting Healthy Public Policy (HPP) [1] or implementing a Health-in-All-Policies approach [1,32,33]. While HPP has been identified as an essential mechanism for practitioners in all sectors to support the health of community residents, HiAP is the recognition that health and health equity are the fundamental responsibility of all levels of government [34]. Adopting an HiAP approach means that all government sectors are required to think about the health impacts of their policies to ensure enhanced “accountability and shared social responsibility for health and well-being” [35], p. 9473. Accordingly, HiAP approaches ensure that there is a prolonged consideration of public health in institutional practices and government policy making [36].

The application of HiAP aligns with healthy community approaches due to shared values and goals (i.e., health and health equity), and emphasizes the need for horizontal and vertical coordination across government departments [37]. This is evident in the creation of laws or regulations by governments concerned with improving community health [32] and the incorporation of a ‘health lens’ into decision making at all levels [38]. But while engagement can occur at all levels of government, HiAP has been most notably implemented at the national and state/provincial levels rather than at the municipal level [33,36,38,39]. According to Mundo et al.’s examination of HiAP in local public health agencies in Colorado, USA, “message framing from the state and public health community is lacking” [36], p. 4, which reinforces policy-makers’ hesitancy to put health on the agenda. Moreover, Ramirez-Rubio et al. [32] identify short political cycles and terms in office as conflicting with the political stability and commitment needed to adopt HiAP.

Municipal governments are optimally situated to adopt health-oriented policies due to their understanding of local needs [18,33,38,40] and context [38], and their direct connection with individuals [33]. Municipalities are also well positioned to address health inequities due to their ability to implement policies consistent with a top-down approach while carefully incorporating grassroots advocacy and knowledge [39]. Further, municipal engagement with HiAP has the potential to subsequently inform the policy directives of higher orders of government. The National Collaborating Centre for Healthy Public Policy is a non-governmental agency in Canada that, among other projects, promotes Health-in-All-Policies as a way for decision makers to consider the health and health equity impacts of their policies [41]. While the organization primarily advocates for HiAP at the federal and provincial/territorial levels [42], it has recently noted that “increased interest in HiAP at the local level, where the leadership is best positioned to operationalize action on the social determinants of health, may influence how the approach is perceived at higher levels of government” [43], p. 7. According to the NCCHPP [42], HiAP approaches involve (1) policy-making processes; (2) collaborative and dynamic solutions; and (3) broad strategies by governments. The NCCHPP [44] uses Vancouver’s Healthy City Strategy as an exemplar for other local governments, particularly in relation to the shared responsibility of sectors and the identification of context-dependent strategy development.

### 1.4. Study Purpose

Municipal governments can create healthy communities and positively contribute to the social determinants of health by leveraging the sectors under their purview [45] to influence the “social environments of daily living” [33], p. 284. But while public policies are the fundamental tools for combatting social problems [46], there is a paucity of research that examines municipal governments’ engagement with HiAP [33,38] and healthy community strategies [37]. As such, the aim of this paper is to interrogate municipal strategies designed to foster a healthy community by identifying the key issues or ‘problems’ local governments attempt to address with these policies. Identifying these conceptualizations of community health will shed light on which social determinants of health are being targeted through municipal initiatives.

## 2. Materials and Methods

### 2.1. Research Design

This study adopts an exploratory qualitative research design [47] to develop practical insights about local governments’ conceptualization of healthy communities. To do so, healthy community discourse is examined in government policy documents from a single research setting representing a typical medium-sized community in Canada’s largest province. Published discourse by municipal governments highlight their priorities and recommended solutions [48], thus exposing the perceived determinants of health and policy responses in the local context.

### 2.2. Research Context and Setting

Canadian census metropolitan areas (CMAs) are home to nearly three quarters (74.4%) of the national population [49]. The Region of Waterloo is 1 of 16 CMAs in the province of Ontario, Canada [50]. CMAs in Canada are defined by their population density and have a population of at least 100,000 with 50,000 or more living in a core area [51]: medium-sized CMAs have between 300,000 and 2,000,000 people [52]. As 1 of 7 medium-sized CMAs in Ontario [50], the Region of Waterloo has a population of 587,165 people [53]. The Region of Waterloo is a two-tiered municipality, with the Region as the upper tier and the cities of Cambridge, Kitchener, and Waterloo and townships of North Dumfries, Wellesley, Wilmot, and Woolwich accounting for the seven lower-tiered governments.

In Canada’s federalist governance system, power and authority is shared by both federal and provincial/territorial governments. Moving to the municipal level, these governments have powers delegated to them by provincial/territorial governments in order to better address the needs of residents in their jurisdiction. For example, in the Province of Ontario, municipal governments are responsible for delivering social services, police/fire services, waste management, and public transportation [54], all of which have impacts on health. In relation to healthy community planning, the Planning Act [55] identifies the responsibility of ministers, councils, planning boards, and the planning tribunal to realize provincial interests, including “the orderly development of safe and healthy communities” (p. 12).

In the Region, 25.4% (n = 147,190) of those in private households identify as immigrants and 27.5% (n = 159,060) identify as visible minorities, while only 12.9% (n = 74,710) of households identify as Canadian in ethnic or cultural origin and 53.1% (n = 307,540) identify with the Christian religion [53]. According to 2018 data on the Waterloo Region, 32.0% of the population is “living with a disability (physical or mental) or chronic illness that limits [their] activity” [56], p. 3. The Region has a diverse range of transportation options, including an integrated bus and light rail system [57], and hosts a mixture of urban, suburban, and rural landscapes [58].

Given the duties bestowed on municipal governments in Ontario and the requirement for them to develop as healthy communities, assessing how local governments manage their responsibility is warranted. Furthermore, as one of several CMAs in the province that is experiencing population growth [49] and maintains a diverse population and urban form, the upper- and lower-tiered municipalities of the Region of Waterloo demonstrate an ideal case to interrogate healthy community discourse.

### 2.3. Data Collection

The primary data sources in this study were municipal plans, policies, and strategies related to healthy communities. In particular, the data sources were located by searching the public websites hosted by upper- (i.e., the Region of Waterloo) and lower-tier (i.e., Cambridge, Kitchener, North Dumfries, Waterloo, Wellesley, Wilmot, and Woolwich) governments. Initially, the first author (KS) used the search function on each website to locate documents that were published by the local government and included the terms ‘healthy community’ or ‘healthy city,’ and then screen all documents for relevance to the jurisdiction by scanning the title and abstract, executive summary, or table of contents; where there was no preamble, documents were considered and screened in the next stage. Once combined, the documents collected from each municipality were reviewed in full to ensure the healthy community content was substantive and of an adequate depth to allow for assessment (i.e., more than a cursory mention of ‘healthy community’ or ‘healthy city’). Only English-language documents published by local governments before 2023 and available online were included in the study. A total of 8 documents met the inclusion criteria and were analysed.

### 2.4. Data Analysis

The healthy community discourse embedded within each document was analysed using Bacchi and Goodwin’s [59] ‘What’s the Problem Represented to Be’ [WPR] approach. WPR dissects the power relations embedded within discourse and assesses priorities from a social justice perspective [60,61]. In particular, the approach illuminates the power within public policy documents by exploring: (1) the issues presented or disregarded from the documents, including an exploration of how power can reinforce or challenge the organization of society; (2) an assessment of the underlying assumptions and how they relate to the problem; and (3) an identification of who is included or excluded from the document [62,63]. Originating through the critique of public health policy [64], the WPR approach has been used to study land use issues, such as strategic planning [65], and local issues, such as chronic homelessness [66], public washrooms [67], and bike sharing [68]. The WPR approach was used in this study to interrogate the ‘problems’ associated with healthy community/city planning.

To analyze the documents retrieved, the ‘healthy community’ or ‘healthy city’ discourse was inductively coded by the first author (KS) through the use of the NVivo 14 qualitative software package. Once the text was coded, the resulting codes were scrutinized using the analytic process prescribed by the WPR approach [59]. The seven-step approach includes the following: Step 1—identify the problems represented; Step 2—appraise underlying assumptions/dualities; Step 3—explore related events; Step 4—consider missing/alternative factors; Step 5—evaluate effects of representations; Step 6—review the production of problems; and Step 7—reflect on information [59]. Discussions of and a reflection on the use of the WPR framework took place during multiple stages of the analysis to ensure consistency with Bacchi and Goodwin [59].

In the current study, *Part One* of the *Findings* section explores the major concerns, underlying assumptions, and relevant events related to each focal area identified as most prevalent across all included documents (i.e., Steps 1 to 3 of the WPR approach). *Part Two* of the *Findings* section, scrutinizes the major concerns for ‘missing’ information and the potential impacts thereof (i.e., Steps 4 and 5 of the WPR approach).

## 3. Findings

In total, 8 government documents from both lower- (6) and upper-tier (2) municipal levels met the inclusion criteria for this study. As outlined in Table 1, the documents examined were published between 2014 and 2022 and included official plans (2), strategic plans (2), and functional master plans including, more specifically, business (2) and transportation plans (2). The most common underlying focus of the healthy community discourse in the documents was the pursuit of economic growth (n = 8) and ecological sustainability (n = 7), which are discussed in depth below.

### 3.1. Part One: Problems, Assumptions, and Events Related to Healthy Community Discourse

#### 3.1.1. Economic Growth

Each document contained discourse related to the economic circumstances of the respective jurisdiction. Three specific concerns related to economic growth were apparent: (1) managing finances, including generating revenue and minimizing expenditures; (2) maintaining space and infrastructure for employment, trade, and access to services, including connecting people to their place of employment; and (3) diversifying economic opportunities and supporting economic development (see Table 2).

The first concern associated with the topic of economic growth was managing the cost of government operations, including service provision and infrastructure development. Several municipalities highlighted the importance of managing and extracting local resources, including goods, to support the economy. The Township of Woolwich [74], particularly, emphasized the potential to generate funds through programming in their recreation facilities in their goal to “improve marketing and promotion efforts for the Township’s recreational facilities and programs to increase participation and related revenues” (p. 25). Related to the topic of managing costs, in some jurisdictions, municipal governments also identified the potential to advocate for or seek out funding from broader jurisdictions or agencies to support new infrastructure or programming. Again, the Township of Woolwich [70] identified that they would “apply for funding for a student arborist” (p. 34) to support their implementation of a local forestry plan, while the Township of Wellesley [73] needed to “research & apply for funding for construction of new recreation facilities” (p. 26). These examples demonstrate the limited funding available to municipal governments and that new initiatives require additional resources for implementation beyond their current capacity.

The second concern related to economic growth was the space and infrastructure needed for employment, trade, and access to services. In particular, the need to maintain industries, such as agriculture or manufacturing, while also welcoming new opportunities in, for example, the education and knowledge sectors was emphasized. This was seen, for instance, in the City of Kitchener’s Official Plan [OP] [14] that identified the need for various industries and employment opportunities to remain within their jurisdiction so that the economic base of the area could be sustained or improved upon. Meanwhile, the Region of Waterloo’s Transportation Master Plan [TMP] [71,72] and the City of Kitchener’s OP [14] both emphasized the importance of ensuring their transportation system was effective in meeting the workforce’s ability to access employment opportunities or workplaces. For example, as part of the TMP, the Region [72] identified the need to “allocate TDM [transportation demand management] funding from capital project budgets to enable delivery of selected residential, workplace, and school measures” (chapter 5, p. 22). The TMP [71,72] also emphasized the importance of ensuring goods could easily be transported in and around the Region.

The third economic growth concern was the ability to diversify current economic opportunities, and utilize or expand existing relationships, which could reduce redundancies, save costs, or generate new sources of revenue. In particular, the need to support local industries and employment opportunities was underscored by the Township of Woolwich’s [74] goal to “collaborate with Affiliated Woolwich organizations to explore opportunities for partnership and program development” (p. 24), which highlights the idea that partnerships could be forged or maintained into the future. Similarly, as identified in the City of Kitchener’s [14] quote in Table 2, the ability to collaborate or provide support for economic opportunities was seen as helping to ensure the viability of the city in future.

Embedded within the broader economic growth priority is the assumption that a stable or growing economy will contribute to a healthy community. However, without acknowledging geographic disparities or social disadvantages related to the statements produced, readers may assume that increased fiscal resources will go to the general municipal revenues.

Municipal governments need to adhere to budgetary limitations without over relying on broader levels of government or external institutions to financially support their continued independence. Specifically, the need for lower- and upper-tiered municipalities in Ontario to balance their own budgets with limited support from broader jurisdictions stems from the enactment of the Ontario Municipal Act in 2001 [76], which mandates that municipalities maintain a balanced budget. Therefore, to expand initiatives, a municipal government in Ontario must rely on revenues generated through the local tax base, mostly through property taxes [52]: this subjects decisions about healthy community initiatives, including efforts to promote health [18], to local economic circumstances and the cost of offering services. While local governments may be able to secure funding from other institutions to help cover costs for specific projects, such as infrastructure development and employment opportunities, municipal decisions risk overlooking the health and well-being of the populations under their jurisdiction by prioritizing opportunities that generate economic growth.

#### 3.1.2. Ecological Sustainability

Ecological sustainability was identified in most of the healthy community discourse reviewed in this study. The two major concerns associated with ecological sustainability were (1) preserving or enhancing natural features; and (2) shifting to more sustainable practices (as indicated in Table 3).

Protecting and enhancing the natural features of an area was evident in the documents given many of the statements related to the viability of green and blue spaces within communities and their utilization by residents. Related to the protection of natural features, specifically, some jurisdictions outlined geographical approaches to maintaining rural, agricultural lands; minimizing the extraction of natural resources; or ensuring environmental management procedures were in place for disasters such as floods or erosion. Related to enhancing the natural features of an area, some documents outlined the need to develop or implement programs for tree and plant life, especially in parks and green spaces, or to educate residents on conservation efforts. For example, the Township of Woolwich [70] identified their collaborative effort to “…educate youth about the importance of trees…” (p. 33) and inform community members about noxious weeds. The City of Kitchener’s [14] OP explicitly links their environmental protection efforts to health considerations. Specifically, policy (6.C.1.2.) under *Section 6: Public Health and Safety* of the City of Kitchener’s OP stipulates that the municipality may require a health impact assessment (HIA), a tool recommended under HiAP [43], to accompany an environmental assessment or a development application.

The second environmental concern, shifting to more sustainable practices, was apparent in documents through the context of both municipal operations and resident actions. Across the documents reviewed, some governments recognized the potential to update equipment and procedures to minimize impacts on the natural environment. In particular, the documents contained examples indicating municipalities could shift towards more sustainable practices, such as using electric vehicles, improving temperature regulation in facilities, or switching to LED light bulbs. Infrastructure developments and updates were also recognized, which included electric vehicle charging stations, implementing green roofs, adhering to LEED standards, and creating compact forms of infrastructure. For example, the City of Kitchener’s OP [14] explicitly identified “the construction of buildings or the retrofit of existing building to LEED standards or equivalent building rating system” (section 17, p.33) as one of several potential community benefits from bonusing provisions.

In terms of resident actions, specifically, several of the strategies identified the desire to support adopting modes of travel that produced fewer emissions. The Region of Waterloo’s TMP [71,72] widely discussed enhancing public transit and active transportation to decrease automobile use. Furthermore, the Township of Woolwich’s [75] OP identified supporting the Region’s efforts of shifting practices by stating that “the Township in conjunction with the Region will support improved air quality through the policies in this Plan that support a more compact, transit-supportive urban form” (chapter 14, p. 2). Of note, the Region of Waterloo’s TMP [72] was the only document with healthy community discourse that explicitly connected ecological concerns to health outcomes in their jurisdiction by stating the following: “13% [of] Waterloo Region residents with asthma, chronic bronchitis, or emphysema,” (chapter 5, p. 13) “12% [of] Waterloo Region residents with cardiovascular disease or diabetes,” (chapter 5, p. 13) and “60% [is] the portion of the types of air pollution, for which transportation is the leading cause” (chapter 5, p. 13).

Embedded within the broader topic of ecological sustainability is an assumption that municipal governments and residents share responsibility for changing their behaviours and equipment, especially in relation to transportation. The commitments and emphasis on using public transportation and reducing vehicle emissions implies that local ecological conditions, such as air quality, will be positively impacted by an increased uptake in sustainable transportation modes.

The requirement to protect the natural environment as an important component of a healthy community can be traced back to provincially enacted legislation, such as the Environmental Assessment Act [77] and the Environmental Protection Act [78]. Within these mandates are policy directives for the “protection and conservation of the natural environment” [78] for the “betterment of the people” [77]. These policies help to contextualize government responsibilities towards ecological sustainability and the subsequent reduction in harmful exposures that impact residents’ health in communities. Despite these mandates, the goals and policies pertaining to ecological sustainability demonstrate a view of shared responsibility, whereby more sustainable practices in operations and of residents, along with the protecting of natural features can have benefit to individuals and populations across municipalities.

### 3.2. Part 2: Missing Components and Impacts of Current Healthy Community Discourse

The existing discourse on economic growth and ecological sustainability relate most clearly to the concept of municipal longevity rather than the experiences of residents who live in communities. More specifically, the economic and ecological foci imply concerns for the longevity of communities in terms of avoiding the need for an amalgamation or dissolution due to economic or climate crises, respectively. Ultimately, the underlying healthy community discourse is not just for current community residents but also for future generations.

As outlined above, the fiscal and procedural requirements related to the economics and ecology of municipalities pertain largely to mandated provincial legislation; however, municipalities are also responsible for “building strong, healthy communities” [55] and should “promote and enhance human health and social well-being” [79], p. 5. Although the economic and ecological environments where people live are widely recognized as determinants of health, an explicit connection between municipalities’ economic and ecological priorities and the health of residents was largely absent in the documents reviewed. The explicit consideration of human health conditions was limited in this study [72] and represents an important ‘missing link’ in the healthy community discourse reviewed. In this study, even though municipal budgets were related to the health of the community, there was no explicit mention of how cost savings and revenue generation would impact residents’ health. This finding is reminiscent of work by Frohlich et al. [80], who note that the exploration of broad determinants of health is not the same as dissecting the pathways through which health is impacted. Explicitly linking healthy community efforts to population health outcomes and illuminating the pathways between policies and the health of the community is necessary for ensuring the effectiveness of approaches and reducing the likelihood of negative unintended consequences [40].

Compounding the absence noted above, few documents presented clear targets or measures related to achieving economic growth and ecological sustainability. With the exception of the Township of Woolwich’s [74] 2023 Corporate Business Plan, which stated that “…the Township has made two aggressive and ambitious commitments to addressing climate change that being the reduction of 80% of our carbon footprint by 2050 and also a 50% reduction by 2030” (p. 5), targets relating priority areas to health outcomes or local health issues were absent. Furthermore, the City of Kitchener’s OP [14] identified that “it is not the intent to develop and include specific monitoring or performance measurement programs as part of this Plan.” (section 17, p.6). However, without goals to implement change and measures of success, evaluations of policies and their impact on health cannot be completed, as identified in Corburn et al.’s [81] work on HiAP in Richmond, California, where a lack of data on neighbourhood health outcomes limited decision makers’ ability to create and monitor indicators to assess change. Given this, municipal policy-makers have missed the opportunity to showcase local economic and ecological circumstances and highlight how their healthy community goals, objectives, and policies may address these concerns. Of relevance, Salgado et al.’s [29] review noted that the prominence of the natural environment as a determinant of health in urban settings was associated with the ability to monitor changes and reach goals. If municipal governments want to take responsibility for the health of residents, it follows that the development of standards or guidelines to assess their health status would be necessary.

This study also illuminated the relevance of multi-scalar governance structures and policy development for healthy communities. In particular, the discourse in Waterloo’s policy documents engaged with laws and regulations created by the Province of Ontario (e.g., the Provincial Policy Statement, Planning Act, and Municipal Act). Similarly, to support their efforts, local governments also referred to other entities (i.e., the Grand River Conservation Authority) with whom they could collaborate. While this shows the multiple regulations and guidelines that municipal governments must consider during policy development, the agencies identified as relevant in this study were limited. In particular, it was noteworthy that public health agencies were not identified or discussed in relation to the priorities identified. This absence may be partially explained by statements in the documents that indicated a displacement of responsibility for the health of community members onto upper levels of government. Specifically, the Township of Wellesley’s [73] assertion that “health is a provincial responsibility but there are local opportunities to promote a healthy living style [sic] and improve mental wellbeing” (p. 23) is illustrative. The disconnect between healthy community efforts and engagement with public health bodies, as well as the offloading of responsibility to other levels of government, is concerning but not surprising given Hancock’s [31] assertion that improving population health “...is not the business that cities are in” (p. 222). Despite this, the lack of recognition and explicit policy guidance on how local economic and ecological environments shape local health concerns puts municipalities at odds with their mandate to consider the public health impacts of their initiatives [79]. As Hancock et al. [25] suggest, this oversight “...seems to stem in part from the understanding that ‘health’ is about health care...” (p. 217).

### 3.3. Limitations

This work has limitations related to the data sources used for analysis. In particular, the documents were located on public, government websites and thus, documents that were unavailable online were omitted from the study. Similarly, versions of documents that have been replaced or updated would not be found through the search strategy used. This means that assessing how healthy community discourse has changed over time was not possible.

The search criteria used to retrieve documents presents limitations. In particular, the scope of data sources included was reduced to those using the term ‘healthy community’ or ‘healthy city’, meaning that ‘community well-being’ or other synonyms of health may have been overlooked, a noted limitation given Hancock’s [25] finding that many initiatives do not use the term ‘healthy community’ or ‘healthy city’ in their name. As a result, if governments felt their initiatives impact the health of communities and residents without stating this explicitly, their position on healthy community planning (broadly) may have been underexplored. Further, while the regional public health institution is a government entity and information produced by them would have been considered through the search tool, the terminology used when conducting the search may have limited the scope of documents retrieved and, therefore, overlooked the specific priorities of public health in the area. A complementary analysis of current public health concerns linked to policy directives would expand on the findings of this study.

While the analysis presented in this work was performed by following a pre-existing framework [59] that has been used by other researchers [65,66,67,68], the initial inductive coding and assessment of priority issues (Step 1 of the WPR framework) was completed by one author (KS). This subjects the coding to one author’s perspective. Although KS has experience using an inductive coding approach, other researchers may have coded information differently; however, the inclusion of representative quotes throughout the *Findings* section and, especially, those found in Table 2 and Table 3 give insight into the type of information found. Further, the authors have acknowledged that the analysis is based on two prevalent issues that emerged from the documents. While other issues could be explored within the same documents, the analysis pertaining to Steps 2–5 of the framework are based on the issues identified, not other information found within the documents.

Finally, this work was based on a small number of documents (n = 8) from one region in the Province of Ontario. The limited number of documents meeting the inclusion criteria may indicate the limited resonance of this terminology with municipal governments, as suggested earlier [25]. Exploring the preferred language and terminology of local governments and analysing initiatives’ alignment against healthy community frameworks may contribute to our understanding of engagement with this approach.

## 4. Conclusions

Through the assessment of major priorities embedded in healthy community discourse from eight documents published from within the Region of Waterloo, the findings revealed that governments have focused on economic growth and ecological sustainability as core components of a healthy community. These focal areas signal a primary concern with the longevity of the respective municipalities; however, there remains a missing link between current healthy community priorities and health outcomes or access to health-promoting resources within the Region. As such, the findings of this study raise concerns about whether local governments have the necessary level of awareness of how their policies relate to population health [32,36,82] and their perceived responsibility for taking action on local health issues [31]. This represents an opportunity for public health professionals to advocate for a greater consideration of local health issues and outcomes among municipal departments. In alignment with the recommendations of Collins and Hayes [40], future research should explore the views of government policy-makers and elected officials with regard to their awareness of and values on health and health equity in their communities.

Municipal governments are required to create ‘safe and healthy communities’ for residents [56]. To reinforce this policy directive, Ontario Provincial Policy Statements from 2005, 2014, and 2020 contained and consecutively strengthened language around healthy community priorities; however, as the findings of this study suggest, the interpretation of this directive may not align with a health promotion perspective. Accordingly, municipal governments are encouraged to take on more responsibility for the health of residents in their communities by explicitly adopting an HiAP approach. The authors return to the NCCHPP’s assertation that adopting an HiAP approach would help to “ensure the macro-social determinants of health and positive health outcomes receive more systematic consideration from policy-makers across sectors” [43]. Through this approach, municipalities are required to identify how a given priority impacts population health and thus how policy development can target health and health equity [33]. To support this process, Lilly et al. [83] recommend additional leadership, championing, and collaboration across sectors when developing healthy community polices. Given their absence from explicit consideration noted above, collaborating with public health departments would be an ideal starting point for municipalities.

Finally, given that municipalities are looking to and incorporating regulations into their strategies, provincial and federal governments can take further action to support healthy community efforts. Although broader levels of government have been reluctant to engage with this approach [25], such engagement would likely help to overcome perceptions that health is solely the province’s responsibility and health behaviours are solely an individual’s responsibility [25]. Furthermore, explicit directives through legislation may begin to overcome the “...political allergy in what are mainly conservative leaning provincial, state and federal governments to recognizing the social, environmental and economic factors that underlie health” [25]. Moving forward, one potential avenue for the Province of Ontario, specifically, is to strengthen language around healthy communities in the Provincial Policy Statement. Further, expanding on the Planning Act’s ‘safe and healthy communities’ guidelines may help municipalities to (re)consider additional determinants of health and local concerns by providing a justification for enhanced action moving forward. Due to the reach of the Planning Act and Provincial Policy Statement, such changes may help to expand the number of municipalities that have healthy community guidelines, addressing any hesitancy due to limited political will among policy-makers that act as a barrier to their adoption [32,36,82].

## Figures and Tables

**Table 1:** Documents containing healthy community discourse.

Document Title	Year	Location	Jurisdiction	Type	Reference
City of Kitchener Official Plan: A Complete & Healthy Kitchener	2014	City of Kitchener	Lower tier	Official Plan	[14]
Woolwich Township Strategic Plan 2020	2015	Township of Woolwich	Lower tier	Strategic Plan	[69]
2017 Corporate Business Plan	2016	Township of Woolwich	Lower tier	Business Plan	[70]
An Overview of Moving Forward the 2018 Transportation Master Plan	2018	Region of Waterloo	Upper tier	Transportation Master Plan	[71]
Moving Forward: 2018 Transportation Master Plan	2019	Region of Waterloo	Upper tier	Transportation Master Plan	[72]
Wellesley Strategic Plan for 2019–2023	2020	Township of Wellesley	Lower tier	Strategic Plan	[73]
2023 Corporate Business Plan	2022	Township of Woolwich	Lower tier	Business Plan	[74]
Woolwich Township Official Plan	2022	Township of Woolwich	Lower tier	Official Plan	[75]

**Table 2:** Economic growth for healthy communities.

Major Concern	Document	Example from Document
Manage finances	Township of Wellesley Strategic Plan 2019–2023	Investigate economic impact and develop a detailed plan to manage and where appropriate promote safer interaction of motorized and non-motorized vehicles for residents and non-residents [73], (p. 24).
	Township of Woolwich Strategic Plan 2020	Improve marketing and promotion efforts for the Township’s recreational facilities and programs to increase participation and related revenues [69], (p. 10).
	Township of Woolwich Corporate Business Plan 2017	Evaluate the potential impacts and benefits of new green energy technology that provides value-added benefits to the local economy while not detracting from quality of life [70], (p. 34).
Space and infrastructure	Region of Waterloo Transportation Master Plan 2019	The Region’s transportation system plays a major role in the Region’s economy. In addition to moving goods to, from, or within the Region, it provides essential connections between residents, businesses, and employees [72], (chapter 2, p. 1).
	Township of Woolwich Official Plan 2022	The Township will, where appropriate, co-locate and integrate public service facilities and public services in community hubs to promote cost-effectiveness [75], (chapter 14, p. 1).
	Region of Waterloo Transportation Master Plan—Overview 2018	Waterloo Region will be a prosperous, sustainable, and healthy community, with viable transportation choices for people of all ages and abilities and for the goods supporting our economy [71], (p. 1).
Diversify and collaborate	Township of Woolwich Corporate Business Plan 2023	Explore alternative revenue generating opportunities for summer/shoulder usage at the Woolwich Memorial Centre [74], (p. 25).
	City of Kitchener Official Plan 2014	The City will continue to collaborate with and support economic development entities in an effort to grow in a manner that provides employment opportunities, supports a diverse economy, and contributes to Kitchener’s future prosperity [14], (section 5, p. 3).

**Table 3:** Ecological sustainability for healthy communities.

Major Concern	Document	Example from Document
Preserve natural features	City of Kitchener Official Plan 2014	We will aspire to build upon our open space system to provide residents with an interconnected and continuous multi-use pathway network and recreational areas while conserving and protecting our natural heritage features. Creating green space in our neighbourhoods for passive and active recreation uses will be a priority as we look to enhance Kitchener’s environment, health and social well-being. We will seek to maximize opportunities for public access to the Grand River to enable its recreational potential to be realized [14], (section 1, p.6)
	Township of Woolwich Corporate Business Plan 2017	Continue to support the activities of TWEEC to promote public awareness of, and education in, environmental enhancement, initiating environmental programs, provide comments to Council on environmental issues, and propose and implement work plans based on priorities identified by the Committee. The Department recognizes the value in preserving and maintaining the many Township green spaces, woodlots and naturalization area and will make a conscious effort to protect such areas [70], (p. 33).
	Township of Woolwich Corporate Business Plan 2023	Preserve and protect passive open green spaces and develop a tree management plan [74], (p. 27).
	Township of Woolwich Strategic Plan 2020	Together with the Region, promote water conservation and wastewater efficiency efforts [69], (p. 11).
Shift to more sustainable practices	Region of Waterloo Transportation Master Plan—Overview 2018	A fast and reliable transit network is essential to support growth and to provide residents a sustainable travel choice. Transit vehicles carry more passengers than cars, and are linked to increased walking trips, and more broadly linked to healthy communities [71], (p. 5).
	Region of Waterloo Transportation Master Plan 2019	Supporting sustainable development is both an economic and environmental goal as it relates to transportation. Projects proposed in the 2018 TMP were selected with both of these aspects of sustainability in mind, aligning with the Region’s sustainable development policies. Specifically, this plan will support sustainable growth in both urban and rural areas while reducing transportation contributions to climate change [72], (chapter 2, p. 1).
	Township of Woolwich Official Plan 2022	The Township, in collaboration with Region, will continue to support initiatives that promote the benefits of reducing energy use, car dependence and idling times, and other initiatives that encourage public agencies, private industries and individuals to participate in energy conservation programs [75], (chapter 14, p. 3).

## Data Availability

The original data presented in the study are openly available at [14,69,70,71,72,73,74,75].
